# Factors Associated With Avoidable Emergency Department Visits in Broward County, Florida

**DOI:** 10.7759/cureus.15593

**Published:** 2021-06-11

**Authors:** Caitlin A Williams, Farzanna Haffizulla

**Affiliations:** 1 Emergency Medicine, Nova Southeastern University Dr. Kiran C. Patel College of Allopathic Medicine, Fort Lauderdale, USA; 2 Internal Medicine, Nova Southeastern University Dr. Kiran C. Patel College of Osteopathic Medicine, Fort Lauderdale, USA

**Keywords:** avoidable emergency department visits, non-emergent emergency department visits, preventable emergency department visits, ethnicity, health literacy, primary care treatable visits, gender, payment and insurance methods, emergency department utilization, race

## Abstract

Background

Improper utilization of emergency departments (EDs) in the United States is an issue that places a large burden on the healthcare system. Previous studies have shown that differences in race, gender, and income level have been associated with avoidable ED visits. Broward County, Florida, is diverse with people from many different socioeconomic backgrounds. The objective of this study is to determine the impact that race/ethnicity, gender, and payment methods have on the rates of avoidable ED visits at hospitals in Broward County, Florida.

Methods

This study utilized a dataset from the Broward Regional Health Planning Council that included ED visits in Broward County in 2019. Secondary data analysis was conducted utilizing a one-way analysis of variance (ANOVA) with post-hoc analysis to compare the proportions of non-emergent, emergent primary care-treatable, and emergent preventable ED visits amongst different race/ethnicities, genders, and payment/insurance methods.

Results

Compared to non-Hispanic white patients, non-Hispanic black and Hispanic patients had higher mean rates of non-emergent ED visits. Women had greater mean rates compared to men for non-emergent ED visits; males had higher mean rates than females for emergent primary care-treatable and emergent preventable. Patients covered by Medicaid had greater mean rates of non-emergent and emergent primary care-treatable visits compared to patients using other payment or insurance methods.

Conclusions

This study identified demographics within Broward County associated with avoidable ED visits. To reduce the burden of ED overutilization on the healthcare system, healthcare providers must better educate the at-risk populations about proper ED use. In addition, a comprehensive assessment of social determinants of health in patients overutilizing the ED will allow for better alignment of resources and policy changes to improve healthcare access and community health.

## Introduction

The emergency department (ED) is a necessary component of healthcare services where urgent and critical health concerns are managed [1]. While many patients who seek care in the ED have conditions that require immediate medical attention, patients also use the ED for treatment of primary care illnesses that could be managed in other settings, such as urgent care centers or outpatient clinics [2]. Furthermore, many patients seek care in the ED for complications and conditions that could possibly have been prevented with proper outpatient care [3]. According to one study, an estimated 13.7% to 27.1% of ED visits in the United States could have been treated at either a retail clinic or urgent care center [4]. A more recent and conservative study found that 3.3% of all ED visits were for conditions that could have been treated elsewhere [2]. Regardless of the approach used to evaluate preventable ED visits, it is clear that there is a need to address this issue.

Avoidable ED visits place a huge burden on the healthcare system, including rising costs [4] and ED overcrowding [5]. It is estimated that preventing unnecessary ED visits could provide a cost savings of approximately $4.4 billion annually in the United States [4]. Furthermore, overutilization of EDs, particularly by patients who could have sought treatment elsewhere, has the effect of increased ED wait times and inefficiency [4,5]. ED overcrowding has been shown to lead to treatment delays and impact patient mortality [5]. Because of the significance of potentially preventable ED encounters, it is important to identify factors that lead to improper ED utilization [5].

Several factors are associated with inappropriate use of emergency services, including age, race, ethnicity, gender, income, insurance coverage, and access to care [6]. With regards to race and ethnicity, non-Hispanic black patients have been shown to use the ED more frequently for non-urgent visits than non-Hispanic white patients [6,7]. Hispanic patients have also been shown to have higher rates of ED utilization compared to non-Hispanic white patients [7,8]. These disparities across racial minorities exist even after adjusting for income level [7]. In terms of gender, studies have conflicting results with some populations experiencing greater inappropriate emergency utilization in men compared to women and vice versa [6].

Poor access to primary care physicians also contributes to non-emergent use of EDs [6]. It has been shown that housing instability and food insecurity are associated with decreased access to primary care, leading to increased ED use and hospitalizations [9]. However, recent expansions in Medicaid coverage have been associated with a decline in ED utilization due to improved access to outpatient clinics [10]. Laws that protect patients’ access to care, such as the Federal Emergency Medical Treatment and Active Labor Act (EMTALA) that allow all patients to be stabilized and treated in the ED regardless of their insurance coverage or ability to pay, have also increased the utilization of the ED by those from different socioeconomic backgrounds because of hospital requirements to provide emergent care [11]. Generally, lower-income patients have higher utilization of the ED for non-emergent conditions [6]. This could partially be explained by the perception that there is payment flexibility in the ED, meaning that patients are not required to make payments upfront, therefore creating less of an immediate financial obstacle [12]. Furthermore, patients covered by Medicare have been shown to have higher rates of ED utilization, even after adjusting for other demographic variables, such as age [13]. These are identifiable factors that contribute to preventable ED visits and warrant further exploration.

Limited health literacy is another modifiable factor that greatly contributes to health outcomes and ED utilization [14,15]. Health literacy refers to an individual’s ability to understand and comprehend health information and services in order to make informed decisions about his or her healthcare [16,17]. In order to make decisions about how and where to seek care, it is imperative that patients understand their healthcare needs, interpret the severity of their condition, and become informed about the options for care available. According to data from the 2003 National Assessment of Adult Literacy, a national study that evaluated the health literacy of adults age 16 and older in the United States, only 12% of adults have proficient health literacy [16]. One study showed that patients with limited health literacy have more than two times the number of preventable ED visits [15]. Another study showed that patients with low health literacy have lower rates of outpatient physician visits, higher rates of ED visits, and higher rates of preventable hospital admissions compared to those with higher health literacy, after adjusting for sociodemographic and health status [18]. However, this gap in health literacy is seen more prevalently in minority populations, with non-Hispanic black and Hispanic adults having lower average health literacy when compared to other racial groups [16].

This study aims to identify demographic factors associated with unnecessary ED visits, defined as ED visits that were either not considered to be emergent or were emergent but could have been prevented or treated elsewhere, in Broward County, Florida. The unique demographic characteristics of Broward County, including people from diverse ethnic and socioeconomic backgrounds [19], make studying this population insightful. This will provide information on where efforts should be targeted to avoid excessive ED utilization with the ultimate goal of properly educating the population about appropriate ED utilization and improving health outcomes.

## Materials and methods

Database

Data from hospital records for the year 2019 were gathered from the Broward Regional Health Planning Council (BRHPC) was utilized for this study for secondary data analysis. The BRHPC, whose goal is to deliver health innovations at the national, state, and local level, receives information from the Florida Agency for Health Care Administration regarding ED visits from hospitals in Broward County [20]. The BRHPC specifically utilizes data from Broward County hospital ED discharges that did not result in inpatient stays. From this database, the system titled “Hospital Inpatient & Emergency Department Analytical System” and the subsection of “Hospital Emergency Department Visits” were utilized.

New York University (NYU) Algorithm for ED visits

The data available through the BRHPC were categorized based on an algorithm developed by the NYU Center for Health and Public Service Research to classify ED utilization [21]. The algorithm was developed to categorize each patient’s visit, based upon initial complaint, presenting symptoms, medical history, age, gender, vital signs, diagnoses, procedures performed, and resources used in the ED, to assign each patient a score for the varying levels of emergent classifications, including non-emergent, emergent - primary care treatable, emergent - ED care needed - preventable/avoidable, and emergent - ED care needed - not preventable/avoidable [21]. Based on the CPT codes of the visit, after placing the patient data into the algorithm, each visit could have a score from 0 to 100 in each category to indicate the appropriate designation. The NYU algorithm has previously been validated demonstrating that emergent visits classified by this system are more likely to result in hospitalization or mortality, compared to non-emergent visits [22].

Inclusion and exclusion criteria

Since the intent was to study ED visits for adults in Broward County, inclusion criteria included patients 18 years of age or older who were Florida residents that visited a hospital in Broward County. The hospitals available for analysis in Broward County include both public and private hospitals, including Broward General Medical Center, Cleveland Clinic Hospital, Coral Springs Medical Center, Florida Medical Center, Holy Cross Hospital, Imperial Point Medical Center, Memorial Hospital Miramar, Memorial Hospital Pembroke, Memorial Hospital West, Memorial Regional Hospital, Memorial Regional Hospital South, North Broward Medical Center, Northwest Medical Center, Plantation General Hospital, University Hospital and Medical Center, and Westside Regional Medical Center. Mental health-related, alcohol-related, substance abuse-related, and injury-related visits were not included in the analysis. Although these patients have health concerns and behaviors that warrant investigation, their unique needs were beyond the scope of this study.

Analysis

Data provided from the BRHPC included demographic information, as well as a percentile component of each ED visit that was categorized by the BRHPC via the NYU Algorithm for ED Visits as non-emergent, emergent - primary care-treatable, emergent - preventable, and emergent - not preventable. For this study, percentage designation of non-emergent, emergent - primary care treatable, and emergent - preventable were of particular importance because the frequency of these types of visits, whether emergent or not, could potentially be reduced through patient education and proper patient management [23]. Therefore, it was important to identify which variables were associated with these types of outcomes. As such, the dependent variables were the frequencies of “non-emergent,” “emergent - primary care treatable,” and “emergent - preventable.” These outcomes were evaluated individually to determine associations between race/ethnicity, gender, and payment/insurance method, our independent variables.

Data analysis was performed using Statistical Product and Service Solutions (SPSS) Statistics version 26 (IBM, Armonk, NY, USA). In SPSS, categorical nominal variables were coded to be analyzed. For each combination of the independent variable and dependent variable, a one-way analysis of variance (ANOVA) was performed, and the assumptions for ANOVA analysis were evaluated. Games-Howell post-hoc analysis was performed to determine the specific demographic categories that had statistically significant differences.

## Results

For the analysis, a total of 264,470 patients were included. Demographics of the sample are included in Table 1.

**Table 1:** Sample demographics of patients visiting an emergency department in Broward County, Florida, in 2019. N = number of patients HMO - health maintenance organization

		N	Percentage
Race/Ethnicity	Asian/Pacific Islander	2,491	0.9
	Non-Hispanic black	117,345	44.4
	Hispanic	55,769	21.1
	Native American	331	0.1
	Non-Hispanic white	82,067	31.0
	Other	6,467	2.4
Gender	Female	162,278	61.4
	Male	102,192	38.6
Payer Method	Medicaid	47,377	17.9
	Medicare	58,131	22.0
	Private, incl. HMO	88,045	33.3
	Self-Pay	55,556	21.0
	No Charge/Charity	8,553	3.2
	Other	6,808	2.6
Age Category	18-39	107,363	40.6
	40-64	98,472	37.2
	65+	58,635	22.2
Hospital Attended	Broward General Medical Center	31,557	11.9
	Cleveland Clinic Hospital	10,599	4.0
	Coral Springs Medical Center	19,902	7.5
	Florida Medical Center	11,765	4.4
	Holy Cross Hospital	16,468	6.2
	Imperial Point Medical Center	11,430	4.3
	Memorial Hospital Miramar	10,592	4.0
	Memorial Hospital Pembroke	12,143	4.6
	Memorial Hospital West	20,001	7.6
	Memorial Regional Hospital	33,270	12.6
	Memorial Regional Hospital South	8,265	3.1
	North Broward Medical Center	17,773	6.7
	Northwest Medical Center	16,396	6.2
	Plantation General Hospital	17,562	6.6
	University Hospital and Medical Center	10,139	3.8
	Westside Regional Medical Center	16,608	6.3
CPT Category	Minor severity	7,310	2.8
	Low/moderate severity	19,073	7.2
	Moderate severity	70,549	26.7
	High severity/non-immediate	112,607	42.6
	High severity/immediate	54,931	20.8

Race/ethnicity

Non-Emergent

There was a statistically significant difference when comparing the different racial/ethnic groups, Welch’s F(5, 3,242.05) = 1,683.32, p < 0.0005. The means of frequencies of non-emergent ED visits are seen in Table 2. Specifically, the mean was greater for non-Hispanic black patients compared to all other races (p = 0.05), including non-Hispanic white patients by 4.77, Hispanic patients by 3.32, Asian/Pacific Islander patients by 3.28, Native American patients by 5.91, and patients of other race by 2.18. The mean for Hispanic patients was greater (p = 0.05) than non-Hispanic white patients by 1.45 and Native American patients by 2.59. Asian/Pacific Islander patients had greater means than non-Hispanic white patients by 1.49 (p = 0.05). The mean was greater for non-Hispanic white patients compared to Native American patients by 1.14 (p = 0.05). Patients with a race of other had a statistically significant greater mean (p = 0.05) than non-Hispanic white patients by 2.58, Hispanic patients by 1.14, Asian/Pacific Islander patients by 1.10, and Native American patients by 3.73. All other comparisons were not statistically significant at the p = 0.05 level.

**Table 2:** Mean NYU ED Visit Algorithm percentages for avoidable emergency department visits in Broward County, Florida, in 2019 by race. NYU = New York University ED = Emergency Department n = number of patients

Race/Ethnicity	Non-Emergent		Emergent - Primary Care Treatable		Emergent - Preventable	
	Mean (SD)	95% CI for Mean	Mean (SD)	95% CI for Mean	Mean (SD)	95% CI for Mean
Non-Hispanic White (n=8,2067)	30.41 (11.73)	30.33-30.49	36.85 (7.57)	36.80-36.90	9.40 (4.27)	9.37-9.43
Non-Hispanic Black (n=117,345)	35.18 (11.96)	35.11-35.25	36.42 (6.58)	36.38-36.46	8.64 (4.45)	8.61-8.66
Hispanic (n=55,769)	31.86 (11.52)	31.76-31.96	37.15 (8.15)	37.08-37.22	8.27 (5.28)	8.22-8.31
Asian/Pacific Islander (n=2,491)	31.90 (21.59)	31.05-32.75	36.55 (17.13)	35.88-37.22	7.75 (10.69)	7.33-8.17
Native American (n=331)	29.27 (27.75)	26.26-32.27	35.29 (20.66)	33.06-37.52	9.96 (19.51)	7.85-12.07
Other (n=6,467)	33.00 (17.78)	32.56-33.43	36.86 (13.13)	36.54-37.18	7.47 (7.81)	7.28-7.66
Total (n=264,470)	32.91 (12.32)	32.86-32.96	36.72 (7.65)	36.69-36.75	8.76 (4.86)	8.74-8.78

Emergent - Primary Care Treatable

The percentage of emergent primary care treatable ED visits showed a statistically significant difference for different racial/ethnic groups, Welch’s F(5, 3,238.53) = 82.41, p < 0.0001. The means of frequencies of emergent primary care-treatable ED visits are seen in Table 2. Non-Hispanic white patients had a higher mean compared to non-Hispanic black patients by 0.43 (p = 0.05). Hispanic patients had a higher mean (p = 0.05) compared to non-Hispanic white patients by 0.30 and non-Hispanic black patients by 0.73. All other comparisons were not statistically significant at the p = 0.05 level.

Emergent - Preventable

There was a statistically significant difference in preventable ED visits for different racial/ethnic groups, Welch’s F(5, 3,238.75) = 505.59, p < 0.0005. The means of frequencies of emergent preventable ED visits are seen in Table 2. Games-Howell post-hoc analysis revealed that the mean increase among non-Hispanic white patients (p = 0.05) compared to non-Hispanic black patients was 0.76, Hispanic patients were 1.13, Asian/Pacific Islander patients was 1.65, and other race patients was 1.93. The means of frequencies of emergent preventable ED visits were also greater for non-Hispanic black patients (p = 0.05) compared to Hispanic patients by 0.37, Asian/Pacific Islander patients by 0.89, and to patients of other races by 1.17. The means of frequencies of emergent preventable ED visits were also greater for Hispanic patients compared to patients of other races by 0.80 (p = 0.05). All other comparisons were not statistically significant at the p = 0.05 level.

Gender

Non-Emergent

There was a statistically significant difference in mean scores of emergent preventable ED for different genders, Welch’s F(1, 190,292.50) = 94,765.30, p < 0.0005. The means of frequencies of emergent preventable ED visits were greater for females than for males, as seen in Table 3.

**Table 3:** Mean NYU ED Visit Algorithm percentages for avoidable emergency department visits in Broward County, Florida, in 2019 by gender. NYU = New York University ED = Emergency Department n = number of patients

Gender	Non-Emergent		Emergent - Primary Care Treatable		Emergent - Preventable	
	Mean (SD)	95% CI for Mean	Mean (SD)	95% CI for Mean	Mean (SD)	95% CI for Mean
Female (n=162,278)	34.26 (11.34)	34.20-34.31	36.40 (6.71)	36.37 - 36.43	8.45 (4.16)	8.43-8.47
Male (n=102,192)	30.77 (13.44)	30.68-30.85	37.22 (8.93)	37.17-37.28	9.25 (5.76)	9.21-9.28
Total (n=264,470)	32.91 (12.32)	32.81-32.96	36.72 (7.65)	36.69-36.75	8.76 (4.86)	8.74-8.78

Emergent - Primary Care Treatable

There was a statistically significant difference in the percentage of emergent primary-care treatable ED visits between the different genders, Welch’s F(1, 173,893.60) = 638.32, p < 0.0005. The means of frequencies of emergent preventable ED visits were greater for males than for females, as seen in Table 3.

Emergent - Preventable

The percentage of emergent preventable ED visits showed a statistically significant difference for different genders, Welch’s F(1, 169,045.45) = 1,472.01, p < 0.0005. The means of frequencies of emergent preventable ED visits were greater for males than for females, as seen in Table 3.

Payer method

Non-Emergent

There was a statistically significant difference in percentages when comparing non-emergent visits for different payment methods, Welch’s F(5, 39,316.89) = 912.28, p < 0.0005. The means of frequencies of non-emergent ED visits are given in Table 4. Games-Howell post-hoc analysis revealed a greater mean among patients using Medicaid (p = 0.05) compared to patients using Medicare with a difference of 3.15 and patients with private insurance with a difference of 1.23. Patients with private insurance had a greater mean than those with Medicare (p = 0.05) with a difference of 1.93. Those who self-pay had a statistically significant higher mean (p = 0.05) compared to Medicaid patients by 1.12, Medicare patients by 1.93, and private insurance patients by 2.35. Patients using other payment methods had greater means than all the different categories of payment/insurance methods (p = 0.05) including Medicaid by 4.13, Medicare by 7.28, private insurance by 5.36, self-pay by 3.01, and no-charge/charity by 3.43. All other comparisons were not statistically significant at the p = 0.05 level.

**Table 4:** Mean NYU ED Visit Algorithm percentages for avoidable emergency department visits in Broward County, Florida, in 2019 by payer method. NYU = New York University ED = Emergency Department n = number of patients

Payer Method	Non-Emergent		Emergent - Primary Care Treatable		Emergent - Preventable	
	Mean (SD)	95% CI for Mean	Mean (SD)	95% CI for Mean	Mean (SD)	95% CI for Mean
Medicaid (n=47,377)	33.65 (11.83)	33.54-33.75	38.41 (7.67)	38.34-38.48	8.78 (5.25)	8.73-8.82
Medicare (n=5,8131)	30.49 (12.05)	30.39-30.59	34.68 (7.04)	34.63-34.74	9.88 (4.38)	9.84-9.91
Private, incl. HMO (n=88,045)	32.42 (11.72)	32.34-32.50	36.72 (5.51)	36.68-36.77	7.45 (3.43)	7.43-7.47
Self-Pay (n=55,556)	34.77 (11.65)	34.67-34.86	37.65 (7.39)	37.59-37.71	9.68 (5.41)	9.64-9.73
No Charge/Charity (n=8,553)	34.35 (15.01)	34.03-34.67	36.74 (9.06)	36.55-36.93	10.74 (7.21)	10.59-10.90
Other (n=6,808)	37.77 (20.05)	37.30-38.25	34.52 (16.72)	34.13-34.92	6.02 (7.29)	5.85-6.20
Total (n=264,470)	32.91 (12.32)	32.86-32.96	36.72 (7.65)	36.69-36.75	8.76 (4.86)	8.74-8.78

Emergent - Primary Care Treatable

There was a statistically significant difference for emergent primary care treatable ED visits for different payment/insurance methods, Welch’s F(5, 39,023.87) = 1,532.18, p < 0.0005. The means of frequencies of emergent primary-care treatable ED visits are given in Table 4. Games-Howell post hoc analysis revealed that the means for patients who used Medicaid were greater than all payment/insurance methods (p = 0.05), including Medicare by 3.73, private insurance by 1.68, self-pay by 0.76, no-charge/charity by 1.67, and other payment/insurance methods by 3.89. Private insurance patients had a greater mean (p = 0.05) than Medicare patients by 2.05 and those with other payment/insurance method by 2.20. No charge/charity patients had a greater mean (p = 0.05) than Medicare patients by 2.057 and other payment/insurance method by 2.22. All other comparisons were not statistically significant at the p = 0.05 level.

Emergent - Preventable

The percentages of emergent preventable ED visits had a statistically significant difference for different payment/insurance methods, Welch’s F(5, 38,645.54) = 3,664.33, p < 0.0005. The means of frequencies of emergent preventable ED visits are seen in Table 4. Games-Howell post hoc analysis revealed that Medicaid patients had a greater mean (p = 0.05) than private insurance patients by 1.33 and other payment/insurance method by 2.75. Medicare patients had a greater mean (p = 0.05) than Medicaid patients by 1.10, private insurance patients by 2.43, self-pay patients by 0.19, and other payment/insurance methods by 3.85. Private insurance patients had a greater mean than other payment/insurance methods by 1.42 (p = 0.05). Self-pay patients had a greater mean (p = 0.05) than Medicaid patients by 0.91, private insurance patients by 2.23, and other payment/insurance methods by 3.66. No charge/charity patients had a mean greater than all payment/insurance methods (p = 0.05), including Medicaid by 1.97, Medicare by 0.87, private insurance by 3.29, self-pay by 1.06, and other payment/insurance method by 4.72. All other comparisons were not statistically significant at the 0.05 level.

## Discussion

Improper utilization of EDs is a concern that places a significant burden on the healthcare system [4]. With improved patient education, these rates have the potential to be reduced. Studies have suggested that the ED may be an appropriate place to educate patients about ED utilization, thereby improving health literacy [15,18]. By identifying demographic characteristics associated with improper ED utilization, this study sought to shed light on this issue in Broward County, Florida. Of note, the clinical relevance of the data described in this study is unknown.

In terms of race and ethnicity, our finding that non-Hispanic black patients had higher mean frequencies for non-emergent visits was consistent with the literature [6]. Furthermore, the fact that Hispanic patients had higher rates of non-emergent visits compared to non-Hispanic white patients was also consistent with the literature [7,8]. However, the results for emergent preventable ED visits contrasted the literature with non-Hispanic white patients having higher mean frequencies than nearly all other races/ethnicities. It is also important to note that the greatest proportion of ED visits in this sample were non-Hispanic black patients (44.4%), although they represent approximately 30% of the population of Broward County [19]. While this discrepancy could be due to many factors, such as the increased prevalence of underlying conditions and limited access to care, providing culturally appropriate patient education to diverse populations about when it is best to use the ED versus the outpatient healthcare setting may help reduce this disparity.

Analysis of gender revealed mixed results, which is consistent with the literature [6]. While females had higher mean rates of non-emergent ED visits, males had higher mean rates than females for emergent primary care-treatable and emergent preventable visits. These varying results make generalized patient education more difficult when looking solely at gender.

While the dataset did not include individual’s income levels, it did include payment methods, including insurance types, which helps shed light on an individual’s financial status. Individuals that are covered by Medicaid qualify for this insurance because they make up a certain income level based on their household size [24]. When taking this into account, the findings that patients covered by Medicaid had greater mean non-emergent and emergent primary care-treatable visits than patients utilizing many of the other different payment methods are consistent with the literature that individuals with lower incomes and those that have Medicaid coverage have higher rates of ED utilization for non-emergent conditions. This relates back to the perception that some people believe that there is more payment flexibility in the ED [12]. Another possible reason why Medicaid patients had higher rates of ED utilization for non-emergent and emergent primary care is that outpatient visits covered by this insurance often require pre-authorization, which does not always allow for immediate outpatient care, while ED and urgent care visits do not require preauthorization [25].

Based on the results of this study, the authors believe that it is essential that patients be educated about how and when to use EDs versus primary care facilities and what conditions warrant emergency care. This has the potential to reduce the number of unnecessary ED visits, which would then in turn lessen the burden on the healthcare system [15]. By identifying demographic factors related to preventable ED visits, efforts to educate patients can be targeted in the ED, as well as in the community. Establishing trusted community health partners is a beneficial way for community members to be educated about proper ED utilization, as well as aid in establishing regular accessible primary care. One study found that ED visits decreased for non-urgent conditions when patients were properly educated by community health workers [26]. These community health workers provided patients with education about utilizing primary care physicians, helped with appointment scheduling, and followed up with patients to discuss any potential barriers that were not previously addressed [26]. Studies like these show the positive impact that educating the public about the healthcare system can have on ED utilization [26]. Furthermore, increasing numbers of urgent care clinics can also serve to treat patients with non-emergent conditions [27].

Efforts in South Florida are currently being made to further educate the community about proper healthcare utilization with the hopes of improving healthcare literacy and health outcomes. One such organization is Hispanic Unity of Florida that works to empower members of the community to become “self-sufficient, productive, and civically engaged” and plays a significant role in health education in Broward County [28]. Specifically, through their “Te Ayudo” program, health navigators help inform members of the community, particularly the Hispanic population, about proper health practices, such as utilizing health insurance and when it is appropriate to use outpatient, urgent care, or ED services [28]. These efforts can be improved upon through further research investigating ED utilization in Broward County.

Future studies

The authors have several ideas regarding how this project can be improved upon. Additional studies with alternative research strategies and statistical analyses should also be conducted. Initially, other demographic variables and social determinants of health should be evaluated to determine their association with ED utilization. Analysis should be extended to other hospitals in neighboring counties and include all age groups. Duplicate studies can serve to validate the findings. In particular, rather than utilizing secondary data analysis, researchers could directly approach patients through a survey prior to discharge from the ED. This survey could include questions about the patient’s symptoms and the perceived urgency of their condition.

Gathering geographic data also has the potential to further impact this research. It would be important to identify where patients who improperly use the ED live to determine if these patients are clustered in certain areas. Furthermore, it is also crucial to determine if patients who overutilize the ED have access to a primary care physician or the transportation necessary to get to regular outpatient visits. 

Future studies should also focus more on the specific diagnoses associated with preventable ED visits. This would provide information about the management of these conditions in an outpatient setting. Since this study utilizes data that has already been stratified by severity of the condition and has been separated from the actual diagnosis, future studies could focus on which diagnoses are associated with a higher rate of ED visits, thus following up with education and healthcare literacy.

Another topic for future studies would be to focus on drug, alcohol, psychiatric, and injury-related ED visits to determine if there are demographic correlations that allow for targeted prevention. While many of these conditions often require serious emergency treatment, proper outpatient management has the potential to reduce ED visits and eventual hospitalization [29,30]. Identifying the populations associated with these conditions in a similar manner to this project has the potential to provide information on how to target education to reduce ED utilization in these groups.

Finally, since the data utilized in this study was from 2019, future studies should focus on ED utilization since the onset of the COVID-19 pandemic. This study can serve as a comparative point to assess how utilization has changed across race, gender, and payment/insurance method given the healthcare implications of the pandemic. Taking a proactive approach to reducing emergency room usage and improving access to primary care is essential. A comprehensive review of relevant determinants of health promotes a holistic approach to improving community health and reducing healthcare costs.

Limitations

While this study utilized a large dataset of valuable information, there were still several limitations to the study. Specifically, because this study utilized secondary data analysis, specific diagnoses were not available for analysis. As such, this study was not able to evaluate which conditions were more likely to be associated with avoidable ED visits; however, this was not a focus of this study.

Another limitation was that patients were divided mainly by race, instead of ethnicity. In South Florida, there are many different ethnicities within racial groups that have unique health concerns, which the study does not address. Further disaggregating this data and future data will shed light on other variables that play a role in ED utilization, such as cultural limitations and patients’ trust in the healthcare system.

It is also important to note that the information provided from ED visits did not include the date or time of service. Therefore, it is necessary to take into consideration that many visits may have occurred after hours when urgent care or ambulatory clinics were closed, creating a greater need for patients to visit the ED. This is of particular importance for visits that were considered to be emergent - primary care treatable.

Finally, there were many outliers in many of the analyses because of unequal sample sizes within dependent variable groups. As such, many of the samples were not normally distributed because of high counts of low and high percentages. Analysis was continued despite these less-than-ideal circumstances. In addition, the inclusion of social determinants of health provides a comprehensive understanding of relevant factors impacting ED usage.

## Conclusions

Unnecessary and avoidable ED visits create a burden on the healthcare system, both regarding resources and financially. Interventions, including culturally appropriate patient education regarding ED utilization, have the potential to decrease this burden. Our study showed that compared to non-Hispanic white patients, non-Hispanic black and Hispanic patients had higher mean rates of non-emergent ED visits. Women had greater mean rates compared to men for non-emergent ED visits, while males had higher mean rates than females for emergent primary care-treatable and emergent preventable. Medicaid patients had greater mean rates of non-emergent and emergent primary care-treatable visits compared to several different payment methods. These findings can help guide patient education to improve health literacy and improve proper ED utilization.

## References

[1] Morganti KG, Bauhoff S, Blanchard JC (2013). The evolving role of emergency departments in the United States. Rand Health Q.

[2] Hsia RY, Niedzwiecki M (2017). Avoidable emergency department visits: a starting point. Int J Qual Health Care.

[3] Hsieh VC, Hsieh ML, Chiang JH, Chien A, Hsieh MS (2019). Emergency department visits and disease burden attributable to ambulatory care sensitive conditions in elderly adults. Sci Rep.

[4] Weinick RM, Burns RM, Mehrotra A (2010). Many emergency department visits could be managed at urgent care centers and retail clinics. Health Aff (Millwood).

[5] Hoot NR, Aronsky D (2008). Systematic review of emergency department crowding: causes, effects, and solutions. Ann Emerg Med.

[6] Uscher-Pines L, Pines J, Kellermann A, Gillen E, Mehrotra A (2013). Emergency department visits for nonurgent conditions: systematic literature review. Am J Manag Care.

[7] Law HZ, Oraka E, Mannino DM (2011). The role of income in reducing racial and ethnic disparities in emergency room and urgent care center visits for asthma-United States, 2001-2009. J Asthma.

[8] Oraka E, Iqbal S, Flanders WD, Brinker K, Garbe P (2013). Racial and ethnic disparities in current asthma and emergency department visits: findings from the National Health Interview Survey, 2001-2010. J Asthma.

[9] Kushel MB, Gupta R, Gee L, Haas JS (2006). Housing instability and food insecurity as barriers to health care among low-income Americans. J Gen Intern Med.

[10] Chou SC, Gondi S, Weiner SG, Schuur JD, Sommers BD (2020). Medicaid expansion reduced emergency department visits by low-income adults due to barriers to outpatient care. Med Care.

[11] Zibulewsky J (2001). The Emergency Medical Treatment and Active Labor Act (EMTALA): what it is and what it means for physicians. Proc (Bayl Univ Med Cent).

[12] Northington WE, Brice JH, Zou B (2005). Use of an emergency department by nonurgent patients. Am J Emerg Med.

[13] Johnson PJ, Ghildayal N, Ward AC, Westgard BC, Boland LL, Hokanson JS (2012). Disparities in potentially avoidable emergency department (ED) care: ED visits for ambulatory care sensitive conditions. Med Care.

[14] Herndon JB, Chaney M, Carden D (2011). Health literacy and emergency department outcomes: a systematic review. Ann Emerg Med.

[15] Balakrishnan MP, Herndon JB, Zhang J, Payton T, Shuster J, Carden DL (2017). The association of health literacy with preventable emergency department visits: a cross-sectional study. Acad Emerg Med.

[16] Kutner M, Greenberg E, Jin Y, Paulsen C (2006). The health literacy of America’s adults: results from the 2003 National Assessment of Adult Literacy. https://nces.ed.gov/pubs2006/2006483.pdf.

[17] Cutilli CC, Bennett IM (2009). Understanding the health literacy of America: results of the National Assessment of Adult Literacy. Orthop Nurs.

[18] Schumacher JR, Hall AG, Davis TC, Arnold CL, Bennett RD, Wolf MS, Carden DL (2013). Potentially preventable use of emergency services: the role of low health literacy. Med Care.

[19] (2021). U.S. Census Bureau QuickFacts: Broward County, Florida. https://www.census.gov/quickfacts/browardcountyflorida.

[20] (2021). Broward Regional Health Planning Council Inc. https://brhpc.org/.

[21] (2021). Faculty & Research | NYU Wagner. https://wagner.nyu.edu/faculty/billings/nyued-background.

[22] Ballard DW, Price M, Fung V (2010). Validation of an algorithm for categorizing the severity of hospital emergency department visits. Med Care.

[23] Morgan SR, Chang AM, Alqatari M, Pines JM (2013). Non-emergency department interventions to reduce ED utilization: a systematic review. Acad Emerg Med.

[24] (2021). Florida Medicaid | Benefits.gov. https://www.benefits.gov/benefit/1625.

[25] (2021). Does Medicaid Require Prior Authorization for Referrals?. https://www.medicare.org/articles/does-medicaid-require-prior-authorization-for-referrals/.

[26] Enard KR, Ganelin DM (2013). Reducing preventable emergency department utilization and costs by using community health workers as patient navigators. J Healthc Manag.

[27] Allen L, Cummings JR, Hockenberry JM (2021). The impact of urgent care centers on nonemergent emergency department visits [PREPRINT]. Health Serv Res.

[28] (2021). Health and Public Benefits | Hispanic Unity of Florida. https://www.hispanicunity.org/content/health-and-public-benefits-1.

[29] Lay B, Kawohl W, Rössler W (2018). Outcomes of a psycho-education and monitoring programme to prevent compulsory admission to psychiatric inpatient care: a randomised controlled trial. Psychol Med.

[30] Reif S, Acevedo A, Garnick DW, Fullerton CA (2017). Reducing behavioral health inpatient readmissions for people with substance use disorders: do follow-up services matter?. Psychiatr Serv.

